# Scientists’ Experiences With Getting Help Making Strategic Communication Choices

**DOI:** 10.1111/risa.70311

**Published:** 2026-07-07

**Authors:** John C. Besley, Sarah Garlick, Alexandra Benitez Gonalez

**Affiliations:** ^1^ Michigan State University East Lansing Michigan USA; ^2^ The Nature Conservancy in New Hampshire Concord New Hampshire USA

**Keywords:** communication infrastructure, environment, interviews, qualitative research, scientists

## Abstract

This article uses semi‐structured individual and focus group interviews to understand scientists’ experiences receiving communication support with a specific focus on the type of help that can enable evidence‐based risk communication decision‐making. Using qualitative thematic analysis, it draws on a primary sample of environment‐focused scientists alongside additional interviews with practitioners (*n* = 46) to explore what might lead scientists to obtain help with tasks such as identifying goals and creating evidence‐based strategies to achieve those goals. We find that the communication support scientists tend to receive is often limited to tactical‐level tasks and that few scientists have had opportunities to work with someone with expertise in helping identify and pursue concrete communication goals.

## 1 Introduction

This manuscript seeks to contribute to our understanding of how scientists studying risk‐related topics in the United States (US) make communication choices. It specifically uses qualitative focus group and individual interviews to try to understand how scientists working in areas related to the environment think about communication support with an underlying analytical focus on understanding what might lead such scientists to seek help with the type of strategic communication thinking underlying risk communication effectiveness (Besley and Dudo 2022b). The rationale for focusing on scientists’ experiences with support is that many scientists working at American universities and other organizations that employ scientists (e.g., government, research laboratories, zoos, and arboretums) have substantial autonomy in deciding what, when, and how to communicate. For example, funders such as the National Science Foundation require scientists to ensure “broader impacts” but provide only broad guidance on how to fulfill such mandates and may not ensure that researchers devote adequate funding to fulfill their broader impact commitments. In turn, the quality and depth of such activities vary substantially (Woodson and Boutilier 2023). That said, despite this autonomy and limited support, US‐based scientists often find themselves—by choice or circumstance—putting time and other resources into communication (Besley et al. 2018; Rose et al. 2020) such that there is potential value in trying to better understand the communication environment within which scientists are working.

The specific focus on strategic thinking reflects past research suggesting limited attention to the strategic element of communication decision‐making. For example, much of the science and risk communication training in the US involves helping individual scientists learn how to speak and write in concise and compelling ways (e.g., Aurbach et al. 2018; Rodgers et al. 2020) and rarely emphasizes long‐term, evidence‐based strategy derived from audience‐specific behavioral goals (Bennett et al. 2023; Dudo et al. 2021). We broadly understand strategic science and risk communication help as the support provided to scientists to assist in the identification and prioritization of concrete, audience‐specific behavioral goals along with reasonable plans to achieve those goals, over time, through intermediate cognitive and affective objectives and tactics meant to affect these objectives (Besley and Dudo 2022b; Hon 1998).

### 1.1 A Brief Defense of Why It Is Important to Understand How Scientists Think About Communication Strategy

One potential concern about a focus on helping scientists think strategically is that it puts the focus on benefiting scientists. However, our understanding of strategy rejects equating being strategic with being self‐interested (e.g., Kessler et al. 2022). Past research suggests that most scientists’ stated goals—at least in the US—are about ensuring that research has a positive impact on individual, organizational, and civic decision‐making rather than self‐enhancement. This is true for both risk scientists (Besley and Schweizer 2022) and other types of scientists studied (Besley and Dudo 2026; Besley et al. 2020). This focus on helping society through communication can also be seen in risk communication scholarship's focus on designing communication efforts using formative research about audiences (e.g., Connelly and Knuth 1998); ensuring public participation in decision‐making to increase the likelihood of decision acceptance, support, and/or decision‐quality (e.g., Kasperson 1986); and crisis/disaster communication (e.g., Greenberg 2022). Ultimately, strategic communication is about being intentional about the identification and pursuit of goals; ensuring ethicality is a separate but related challenge (Garlick et al. 2025).

Our focus on understanding scientists’ experience with getting communication help also rejects the idea that the purpose risk communication is simply to help people make decisions such that it is inappropriate for scientists to think about behavioral goals (e.g., Fischhoff 2007). Arguably, such perspectives do not adequately account for the reality that (a) many risk scientists want people to consider specific behaviors (i.e., take mitigating or protective action, fund science) and that (b) encouraging audiences to “consider scientific information” when making decisions can itself be understood as a behavioral goal. In this regard, scientists who “just” want to help people make decisions can benefit from thinking about their audiences’ behavioral contexts (i.e., the behavior about which a decision may need to be made). Ultimately, the type of information a scientist might share if they wanted to help “people consider relevant scientific evidence when making decisions about getting vaccinated” is similar to what they might share if their goal was for “people to get vaccinated” (e.g., information about risks and benefits, norms, efficacy, and key‐actor trustworthiness). Finally, those who reject strategy as being antithetical to contemporary norms around the value of two‐way communication similarly miss the reality that communicators can use strategic thinking to design communication where the goal is to affect their own behavior as a function of updating their own beliefs (i.e., genuine consultation) (Besley and Downs 2024a).

### 1.2 The Current Research

The research shared here started from the premise that there are several potential reasons—described in the literature review below—for why scientists may not be getting the type of strategic communication help that other actors in society receive when they make communication decisions. This project was thus partly an effort to obtain empirical evidence aimed at better understanding the current landscape of how scientists are experiencing and thinking about professional communication support with the idea that facilitating such conversations would provide insight into what might lead scientists to get strategic support. Although most science and risk communication research focuses on audiences, the current work is part of a broader focus on understanding how scientists think about communication with the expectation that doing so can help enable higher quality communication choices (e.g., Vazquez et al. 2021).

Below we make our research questions explicit and then elaborate on three potential reasons presented in the context of recent attempts to theorize scientists’ communication choices as behaviors that can be understood using concepts from theories of planned behavior and trust. From there, we describe the interviews and provide results organized into three themes that we think capture how our interviewees thought about the support they receive. Section 5 suggests a potential path forward.

The work focuses on scientists from environmental science–related fields both because of a desire to put an initial focus on a subject where there are opportunities to improve risk‐related communication effectiveness and based on the research team's area of expertise. Our expectation is that scientists in other areas might provide similar answers, but this is a topic for future research.

## 2 Literature Review

### 2.1 What Does It Mean to Get “Help With Strategic Communication”?

As noted, an initial premise of the current work is that most environmental scientists are willing to communicate through face‐to‐face, online, or news media channels (Besley et al. 2018). Moreover, such willingness exists despite the reality that many US scientists have few individual‐level incentives (e.g., recognition in tenure and annual review processes) to spend time, funding, and other resources on communication (Ecklund et al. 2012; Kimbrell et al. 2022).

The second major premise of the current work is that scientists who communicate face a wide range of decisions about what information to share and seek, such that they may need help from groups such as professional communicators. This reality underlies our focus on understanding scientists’ experiences with getting communication support. For example, trust‐related theory suggests that scientists who have a goal of increasing the likelihood that people turn to scientists for advice on risk‐related questions (i.e., behavioral or relational trust as a goal) may need people to see scientists as trustworthy, including high in ability (i.e., expertise), benevolence (i.e., caring), and integrity (Hendriks et al. 2015). A strategist would thus help a scientist identify the person or group of people with whom the scientist wants to have a more trusting relationship and help develop a plan (i.e., strategy) for building that relationship. We know little about the type of advice that scientists get from communication professionals, but the aforementioned research on science communication training programs (Dudo et al. 2021), at least, suggests that few trainers provide scientists with this type of theory‐based guidance and instead focus largely on tactical advice such as avoiding jargon and using narratives.

This focus on tactical advice echoes a distinction that the strategic communication literature makes between practitioners who provide strategy support and those who provide technical or tactical support. Building on early work on public relations roles (Broom and Dozier 1986), Excellence Theory researchers have argued that a key factor that leads to “excellent” organizational communication is a well‐trained communication leader who focuses on communication strategy in dialogue with organizational executives responsible for overall organizational strategy (Grunig et al. 2007). Part of strategy, in this regard, includes helping organizations understand potential interlocutors and ensuring that organizations are meaningfully responsive to outside voices (i.e., what science and risk communicators interested in public engagement might see as meaningful, two‐way dialogue (Trench 2008)).

Although the science and risk communication literature has paid only limited attention to strategic communication scholarship (Van Dyke and Lee 2020), this differentiation between strategic and tactical support provided a useful distinction for our attempt to understand scientists’ experience with getting help and moving towards more evidence‐based communication (Jensen and Gerber 2020). Experiences with strategy help, in this regard, might look like interactions that go beyond help writing press releases or producing graphics or videos. It is the type of help that could support scientists trying to decide whether creating content makes sense and what might go into that content. It is equally the type of help that might allow scientists to prioritize hearing others’ perspectives so that they can update their own thinking and behavior. Unfortunately, we know little about what leads scientists to obtain such help or their experiences with the help they receive.

The following section shares the conceptual framework and logic that guided our data analyses.

### 2.2 Explication of Potential Reasons That Scientists May Not Seek Strategic Help

#### 2.2.1 Using Behavior Change and Trust Theories to Understand Scientists Help‐Seeking Decisions

Social science research provides substantial guidance into how to think about why someone might choose to engage in any given behavior. The current study is thus partly built on the idea that we can study scientists’ decisions about whether to seek out strategic communication support using concepts from theories such as the Theory of Planned Behavior that seek to understand behavioral choices (e.g., Fishbein and Ajzen 2010; Montano and Kasprzyk 2015). Treating the decision to seek strategic support as an intentional, planned behavior means that interviews with scientists aimed to elicit insight into the degree to which they believed that communication support—and especially strategic support—is more beneficial than risky, normatively acceptable and expected, and feasible given available resources and skills. In other words, rather using a fully grounded approach, we use concepts from past quantitative research as sensitizing concepts (Bowen 2006) to help us make sense of scientists’ views about communication support. This approach builds past quantitative research focused on understanding scientists overall willingness to communicate (Poliakoff and Webb 2007), as their choices about goals, objectives, and tactics (Besley and Dudo 2022a). Such work generally suggests that scientists choose behaviors when they believe the behavior is likely to be beneficial (i.e., useful, enjoyable, satisfying) and feasible (i.e., they have the skills and resources).

Beyond ideas from behavior change, the current study also recognizes that the decision to seek communication support involves making oneself vulnerable to the people who provide the support. This idea of making oneself vulnerable to another actor represents the core idea of what constitutes behavioral trust in the types of trust theories (e.g., McCroskey and Teven 1999; Schoorman et al. 2007) that risk communication researchers use to understand decision‐making in situations of uncertainty (Besley et al. 2026). Similar to behavior change theories, such research shows that people put their trust in others as a function of specific evaluative beliefs. Although the specific belief labels vary, this might include beliefs about an expert's ability (i.e., expertise), benevolence (i.e., goodwill), and integrity (i.e., honesty, values). For the current project, this meant that we also sought to use our interviews to understand scientists’ views about professional communicators and used trust‐related concepts as sensitizing concepts (Bowen 2006).

Together, the focus on using concepts from long‐term behavior change and trust research can be summarized using the following research questions. The first highlights our interest in understanding how scientists think about communication support, and the second recognizes our desire to better understand the nature of such beliefs.

RQ1: What beliefs, if any, appear to lead scientists to seek or receive strategic communication help?
RQ2: What experiences, if any, appear to shape scientists’ beliefs about strategic communication help.

The next subsection describes what we thought we might hear based on the limited available research as well as both logic and our own experience as researchers and practitioners.

#### 2.2.2 Reason Scientists May Not Seek Strategic Help #1: Scientists Have Not Formed Beliefs About Asking for Strategic Communication Help and Thus Cannot Logically Ask for Help

RQ1 asks about what beliefs might lead scientists to seek strategic communication help, but scientists who do not know a behavior exists cannot logically have the types of positive beliefs about the behavior that behavior change research (e.g., Montano and Kasprzyk 2015) suggests leads to behavioral willingness.

As suggested above, one reason we think it is possible that many scientists may not think about science and risk communication in terms of strategic communication is that past research suggests that few trainers focus on strategy and instead focus on media interview training and producing journalism‐like videos and writing (Dudo et al. 2021). Similarly, looking at the roster of past speakers for events such as ComSciCon (2026)—a long‐running, multi‐site student‐run series of conferences on science communication in the US—suggests that it typically features more journalists and content creators than communication strategists or social scientists (see also: Baron 2010). We also think it is noteworthy that two recent studies of university communicators in Switzerland suggest that these communicators largely focus on helping universities produce content and achieve university goals—often with a focus on news channels—and give only limited attention to helping scientists strategize around achieving goals that build from their research (Fürst et al. 2024; Volk et al. 2023). Of course, there is a long history of studying scientists’ experiences with the news media (e.g., Marcinkowski et al. 2014; Peters et al. 2008), but what is key for the current study is a recognition that a focus on helping scientists think in evidence‐based and strategic ways is relatively new (Jensen and Gerber 2020; National Academies of Sciences, Engineering, and Medicine 2017).

In sum, although this project focuses on understanding how scientists think about strategic communication help (RQ1), it was important to remain open to the idea that most scientists will have had few experiences (RQ2) with professional communicators other than journalists and thus have few substantive behavioral beliefs about strategic help. This further means that they will not have strong positive (or negative) beliefs. Such a finding might suggest that researchers who want to further understand scientists’ views about strategic communication help may need to create opportunities for scientists to receive such help so they can begin to develop positive beliefs.

#### 2.2.3 Reason Scientists May Not Seek Strategy Help #2: Scientists Would Like Help With Strategy but Cannot Access Such Help Because They Lack the Resources

A second reason that scientists might not seek help with communication strategy is because they believe—perhaps correctly—that they lack the resources needed to access such help. This somewhat presupposes that scientists know that strategic help exists and have formed somewhat positive beliefs; (RQ1)—which, as discussed above, may not be true—but it could also be a matter of degree. For example, it might be that many scientists have limited general beliefs about communication strategists and would be happy for help; they just do not have strong beliefs about communication “strategy” beyond the sense that such specialized help is not something they can readily access due to cost, availability, or their own skills. From a behavior‐change perspective, this might be understood as negative behavioral control beliefs (Montano and Kasprzyk 2015).

The only study we found related to this topic suggested that environmental scientists who believed that their research site had the resources to develop an engagement strategy were somewhat more likely to support developing a strategy while also being fairly neutral on the degree to which they believed their site had such resources (Besley and Downs 2024b). This study further found that, when asked, scientists indicated positive beliefs about having an engagement strategy but expressed only middling beliefs about the degree to which they personally had the skills needed to contribute to creating such a strategy. Recent qualitative work suggests that environmental scientists value the support of communication professionals and leadership when they can get it (Besley et al., 2021). Older qualitative research that lamented a lack of communication support (Gascoigne and Metcalfe 1997) is also consistent the focus here on assessing the degree to which scientists may not be seeking help because they do not believe that such help is feasible.

More broadly, such research is consistent with survey work on scientists’ overall willingness to participate in communication activities that suggests a lack of perceived time was a consistent and relatively strong negative correlate of communication activity (Besley et al. 2018) and interview work suggesting that scientists see communication as difficult (Davies 2008). It is also consistent with a general interest by communication trainers and evaluators in fostering scientists’ self‐efficacy to enact specific communication activities (e.g., Rodgers et al. 2020; Stylinski et al. 2018). It is noteworthy, however, that much of the focus on self‐efficacy focuses on skill‐based self‐efficacy (i.e., scientists’ confidence in their ability to tactical skills such as communicate clearly) and not “behavioral control”‐based self‐efficacy (i.e., issues of access to resources such as time, money, and support) (Montano and Kasprzyk 2015). Research highlighting the benefits that can come from providing scientists with communication support may also be relevant (e.g., Driscoll et al. 2011).

Looking forward, one reason to learn more about the degree to which science and risk communicators believe they can obtain meaningful help with strategy (RQ2) is that one path forward (RQ1) might be to help scientists think through how they could reallocate existing resources towards strategic help or identify other paths to obtaining such help (RQ1).

#### 2.2.4 Reason Scientists May Not Seek Strategic Help #3: Scientists Have the Ability and Resources to Ask for Strategy Help but Do Not Trust That Engagement Professionals Will be Adequately Helpful, Given the Costs

A final potential reason that some scientists might not seek help is because they do not (a) have positive beliefs about the value of strategic help and/or (b) that they do not believe those people who would help are trustworthy. In other words, they have negative beliefs about strategists and the help they would provide (RQ1), possibly based on past experiences (RQ2). Trust, in this regard, could be understood as a willingness to make themselves vulnerable to a strategist in the form of taking advice (or committing resources to advice‐seeking), and trustworthiness perceptions might include beliefs about the available professionals’ abilities (i.e., expertise), benevolence (i.e., motivations), and integrity (i.e., shared morals, honesty) in the context of providing advice (Hendriks et al. 2015). Relatedly, scientists might not believe interacting with strategists would be enjoyable or useful. They might even believe doing so is risky, either in terms of lost access to resources or normative sanctions (i.e., their colleagues might look down on them) (Montano and Kasprzyk 2015).

Qualitative research noted above suggested that scientists’ experiences and views (RQ1) about communication practitioners helped facilitate increased reliance on those professionals (Besley et al., 2021). In addition, the cited survey research on scientists’ views about engagement strategy also found that research site leaders who wanted communication professionals to take a lead on communication activities were also more likely to support the development of a collective communication strategy (Besley and Downs 2024b). Also relevant are several studies that have begun to explore how university‐based communicators conceptualize their role (Fürst et al. 2024; Volk et al. 2023) as such role conceptions likely affect how scientists, in turn, perceive those who might potentially help them with strategy. Entradas et al. (2023), for example, noted that many university communicators focus on administrative communication (e.g., crisis communication, fundraising, reputation management, student recruiting), rather than communication related to scientists’ priorities. It seems reasonable to expect that believing that communication experts prioritize university goals rather than scientists’ goals might lead scientists to lack trust in communication experts.

Looking forward, data that speak to how scientists perceive practitioners as a function of positive or negative experiences (RQ2) could indicate whether more needs to be done to build trusting, interdependent relationships (RQ1) between scientists and science/risk communication practitioners.

### 2.3 Summary

Limited research seems to have focused on the nature (RQ1) and origins (RQ2) of scientists’ views about communication support and the types of help practitioners might provide. However, we believe there is a compelling reason to try to better understand these topics with a specific focus on the degree to which scientists’ experiences include strategic support.

## 3 Methods

### 3.1 Sample and Interview Implementation

Our sampling goal was to gain access to US‐based environmental science‐minded researchers and practitioners with an interest and experience in engagement. We focused on scientists with an interest in environmental issues out of a desire to ground the work in a topic where there is a full range of communication goals (Besley et al. 2020). This topic also fit with the author team's experiences and interests. Although our research questions focus largely on scientists’ perspectives, we also expected that including some practitioners could provide additional insight into how they think scientists are thinking, as well as insight into the degree to which their discussions are consistent with scientists’ perspectives. In practice, most of the practitioners had science backgrounds, and their responses generally seemed similar to scientists. Initial sampling occurred through science/environment and society listservs and social media for the annual meetings of the American Geophysical Union (AGU) and American Association for the Advancement of Science (AAAS). Additional sampling was then done through the emails to US‐based corresponding authors from March 2021 to March 2023 of *Frontiers in Ecology and the Environment* and *Biosciences*. Both are highly cited academic‐society‐owned journals where scholars with an interest in engagement and the environment might publish. We ended up with 46 participants with whom we conducted focus groups (*n* = 38) and interviews (*n* = 8). The use of individual interviews, especially later in the project, reflected a desire to build on the scientist group interviews with deeper conversations and our sense that the focus group format was not proving integral to eliciting scientists’ experiences and perspectives. Specifically, our sense was that scientists’ perspectives and experiences were such that, although participants could engage in meaningful group conversation, the conversations themselves were not generating additional insights beyond what might be obtained through individual interviews.

The second author (a practitioner with substantial moderation experience) led initial face‐to‐face group and individual interviews at the annual meetings of the AGU (December 2022) and the AAAS (February 2023). The first author also participated in these discussions. The second co‐author also led online group interviews with the co‐author in Spring 2023. The second co‐author stepped back from the project in March 2023 when she left an academic‐oriented organization for a non‐governmental role. After that point, the first co‐author led all interviews except two (these were led by a graduate student, the third author).

- Focus group interviews with four groups of practitioners (*n* = 8)
- Focus group interviews with 11 groups of scientists (*n* = 28)
- Individual interviews with scientists (*n* = 8)

As qualitative work with a small, non‐probability sample, we intentionally avoid connecting responses to specific respondent characteristics, although Table 1 provides an overview of sample characteristics by demographics and we note cases where a response seems linked to a specific context. Similarly, almost all the scientists interviewed were in some type of environmental science/ecology‐related subfield with various types of overlap (e.g., biogeochemistry, oceanography, and conservation biology) making it infeasible to separate responses by field. That said, two of the scientists were social scientists studying environmental topics.

**TABLE 1:** Sample characteristics.

	All (*n* = 46)	Scientists (*n* = 36)	Practitioners (*n* = 10)
Woman	29 (63%)	21 (58%)	8 (80%)
White	34 (78%)	28 (78%)	6 (60%)
Black	3 (7%)	3 (100%)	0 (0%)
Asian	4 (9%)	2 (6%)	2 (20%)
Hispanic	4 (9%)	2 (6%)	2 (20%)
Student	4 (9%)	4 (11%)	0 (0%)
Junior	12 (26%)	8 (22%)	4 (40%)
Mid‐career	12 (26%)	8 (22%)	4 (40%)
Senior	18 (39%)	16 (44%)	2 (20%)
University/Research institute	34 (74%)	29 (81%)	5 (50%)
Government	5 (11%)	3 (8%)	2 (20%
Association	3 (7%)	2 (6%)	1 (10%)
Museum/Informal science	5 (9%)	2 (6%)	2 (20%)

### 3.2 Interview Structure

The individual and focus group interviews were set up as semi‐structured conversations with the expectation that it would often make sense to explore topics raised by participants in more detail (Kvale and Brinkmann 2015) (see Table S1 for the interview guide). In both cases, we began with questions focused on eliciting positive and negative experiences (i.e., tell stories; Seidman 2006, 89–90) working with communication professionals (or scientists), followed by a question that sought to understand the degree to which interviewees believe they understood how practitioners (or scientists) think about communication. This was followed by a direct question focused on when scientists should seek communication help. A set of questions then sought to assess what proportion of a project budget should typically be devoted to communication and who they would hire on a communication team. The interviews concluded by asking respondents about their perceptions about communication research needs. One noteworthy aspect of this focus was that our questions focused on overall experiences and did not specifically focus on strategic help. This was done because we wanted to allow respondents to describe their experiences and priorities without specifically emphasizing the strategic element. That said, the strategic element arose during probes. For the focus group interviews, a moderator encouraged respondents to build on other participants’ responses while seeking to both ensure that no participants dominated the conversation and all participants were able to contribute despite potential issues of power (Smithson 2000). As noted above, however, we ultimately switched to individual interviews near the end of the project based on a sense that the challenge of moderating a group conversation was providing limited benefits relative to the challenges of scheduling and having dedicated time to talk with any single individual.

Respondents completed an informed consent form consistent with the lead author's university and associated legal requirements.

### 3.3 Coding

Using ideas from reflexive thematic analysis, following initial data familiarization stage, initial codes were constructed during open coding using the theories discussed above as sensitizing concepts (Bowen 2006) to help categorize different ideas/topics heard during interviews. Key theories included the aforementioned research on Excellence Theory (Grunig et al. 2007) and scientists’ communication choices (e.g., Besley and Dudo 2022a). This coding was done in NVivo. The codes were then used to identify and name the primary themes discussed below (Braun and Clarke 2022). The specific codes highlighted below using italics represent only key codes used and do not emphasize the three overarching themes that were developed on the basis of these codes.

No effort was made to quantify the frequency with which specific codes were used. Such quantification would have little meaning given the qualitative nature of the analyses and the underlying sample. However, data summaries were used to attend to how often codes were used across interviews and within interviews. Separately, the third author developed brief summaries of each conversation to enable discussions around the themes as part of a multiphase effort to come to familiarize ourselves with the data, generate working codes, construct and review potential themes, and write this report (Willig et al. 2017).

Internal validity was sought based on (a) the authors’ own participation in the interviews, (b) a multiphase process of reading and coding interviews, (c) post‐coding review of the coded content, and (d) discussions that occurred related to the interviews by the research team. Direct quotations are used below to illustrate key ideas. The analyses were further grounded through (e) discussion of summary findings with an expert advisory board. This group consisted of three practitioners and three researchers with expertise on topics related to the work who had existing relationships with the research team such that they were comfortable providing guidance and critique. We did not attempt to differentiate between what we heard in the interviews and focus groups, but our general sense was that any differences were minor in the context of the current research questions.

### 3.4 Positionality

The primary issue of positionality that may have affected the project is that the authors share a history of seeking to advance strategic thinking within science and risk communication research and practice. In addition, the first two authors are fortunate to have had professional positions that have allowed them to explore such ideas in ways that could have created overly high expecations about what is feasible for communication practice within current science infrastructures. Such professional positions, as well as some demographic characteristics that correspond with groups groups who have not been historically marginalized, may have also have made it more difficult for them to recognize and fully understand issues of power and privledge as experiencedy by interviewed scientists and practitioners.

## 4 Results

Following initial open coding and discussion about the data, both among the co‐authors and an external advisory board, the authors decided that three overarching themes could help make sense of the data in the context of the two research questions. These themes were

1. Communication strategy is RARELY TOP‐OF‐MIND;
2. Scientists (mostly, still) see communication as research PROMOTION; and
3. Scientists APPRECIATE any help they can get.

We discuss each of these themes in the context of our research questions and the specific coding underlying our decision to suggest these themes. Capitalized text is used to identify themes below, whereas the codes underlying the themes are italicized.

### 4.1 RQ1: What Beliefs, If Any, Appear to Lead Scientists to Seek or Receive Strategic Communication Help?

Overall, this could be seen as a straight‐forward question to answer because we did not find substantial evidence that most scientists have strong or deep existing beliefs—whether positive or negative—about getting strategic communication help (RQ1). This is especially noteworthy since an interest in communication likely contributed to scientists’ decisions to participate in the research. As discussed below, this finding reflects the theme about strategy being RARELY TOP‐OF‐MIND. However, we also found that many of the people with whom we spoke seemed to hold beliefs consistent with seeing science and risk communication as either the broad PROMOTION of research (often coded as *goal: promotion/media relations*) or some form of effort to “dispel belief” (INT6_sci_) or “build support” for science (FG2_sci_). For example, one communication professional focus group participant talked about how their organization had good infrastructure for communication as function of having people who “are there, basically for outreach, and for communicating our science through video, various media” (coded as *goal: promotion/media relations*) (FG4_pro_). This type of response is noteworthy because it suggests that the benefit of communication is largely as PROMOTION in the form of an effort to deliver science content *to* people and not as activity meant to achieve behavioral goals through communication.

This finding that strategy help was RARELY TOP‐OF‐MIND was especially evident when participants were asked to think about who they would like to have on a communication team. Responses generally pointed towards the benefits of having access to tactical skills—writing, social media, design, and visual content creation—on their team (coded as *desired role—tactical*) while only occasionally indicating that they might benefit from help with strategic tasks such as identifying goals and strategies to achieve those goals (coded as *desired role—strategic*). When participants indicated that they would want someone who leans towards strategy, it was typically someone with an expertise in community relations or justice issues who could help them build relationships with marginalized groups (coded as *desired role—strategic—community relations/justice*). For example, in this quotation, the interviewee initially talks about media skills but then transitions into saying they want someone with audience knowledge and an ability to push back against scientists.

I definitely would want somebody who has experience with public relations, I would want somebody who has a knowledge of the community being served. … And I would also want somebody who, in both of those people, I would want people who had the same level of entitlement about their own skill sets, as the scientists have about theirs. So they can, you know, face off in a room with them. (INT1_sci_)

Our sense that the interviewed scientists and practitioners RARELY had strategy TOP‐OF‐MIND and think about science and risk communication mostly in terms of PROMOTION is consistent with the idea that interviewed scientists did not have strong established beliefs about strategic communication help. That being said, our third theme is the idea that scientists APPRECIATE any communication they can get, and this could be seen when we described the idea of a science communication strategist to the participants. At that point, scientists frequently indicated they would enjoy access to such help, if they believed they could access it (coded as *strategist—would be nice*). A similar proportion, however, also indicated that it was scientists themselves who would have to play the communication strategist role (coded as *strategist—scientists’ role*). Overall, there seemed to be a sense that getting strategic support was unlikely due to access and cost. In the language of behavior change, these could be understood as negative behavioral control beliefs.

Another way that interviewees seemed to speak to about getting help with communication strategy was in how they responded to questions and probes about what research could be done to help the scientific community be more strategic in their communication efforts. One of the most common responses was that respondents said that there was a need for more research on training on how to train scientists to communicate (coded *research needs—better training*). In other words, when asked about help, there seemed to be a tendency to think in terms of helping scientists do more communication themselves rather than help aimed at reducing the burdens of communication. A similar proportion of interviews included content that focused on what interviewees saw as a need for more research that helps scientists evaluate impact. In some cases, this was coupled with a need for a desire for strategic help (often from social scientists) to aid with evaluation. Again, however, discussions of evaluation often focused on the need to demonstrate successful research PROMOTION to enable requests for funding.

A final way interviewees seem to be thinking about communication help—including strategic help—was a belief that communication was not something that scientists should (or could) be expected to pay for from research funds, especially for relatively small grants. When asked about where funding should come from, respondents often indicated that either their universities or funder supplements should pay for communication support (coded as *funding‐external funds* and *‐internal funds*).

[T]he university should fund and they do to some extent, but they should probably fund more direct communication and outreach and public engagement stuff. [It] could be baked in just to the overhead. I don't know. I mean, it would be great if the federal government was just like, we're going to pay for this. But obviously, that's not going to happen. INT3_sci_

On the other hand, a few scientists recognized that funding opportunities are limited and thus believed that communication was an activity that they needed to work to make happen through collaborations and sharing of resources (coded and *‐in‐kind support*), rather than reallocating funding from research budgets. One respondent highlighted the various places their organization cobbles together resources for communication.

We just sort of leverage it out, however, and wherever we can, if it's through a grant or if it's because someone's coming to us to do it for us. And we are using in‐kind support to make it happen. But we don't, there's not enough room in our budget … NSF should hear that it should be something that we would all love to do if there was more ability to budget for it … FG09_sci_

Scientists who had grant funding often noted that they paid a substantial amount of their grant to their universities (i.e., “overhead,” in the US) and that their employers should use some of this money to help them communicate. One thing to note about this desire for help is that it conflicts with the idea that communication is something that scientists should be responsible for doing (and be expected to seek training for) and suggests that scientists are open to the idea of communication as something they do in collaboration with their organizations. Indeed, the idea that scientists APPRECIATE professional communicators who take on shared responsibility for communication was something we heard in many of the interviews (coded as *collaboration–work with professionals)*.

I've also had really good experience with the institution that I work at and the communications team, and I think there's some like, relationship there, they kind of for me, they know who I am. They know what my focus is, they know what my audience is. And so it doesn't take much for me to give them a little bit of information that they can kind of run with. FG04_sci_

#### 4.1.1 Summary of RQ1

Although we explore the topic in more detail in the discussion, our findings for RQ1 are most consistent with “reasons” #1 and #2 for why scientists may not seek strategic help with communication. Specifically, the fact that strategy help is RARELY TOP‐OF‐MIND and that communication is generally seen as PROMOTION seems consistent with the idea scientists may “not have the knowledge or skills needed” to ask for strategic communication help (i.e., Reason #1). Further, the concerns respondents raised about limited funding alongside the fact that scientists APPRECIATE communication help point to the idea that scientists may limit asking for communication help because they believe—possibly for good reasons—that they face resource constraints (i.e., Reason #2). In contrast, we heard little about a lack of trust in communication professionals or the value of communication help (i.e., Reason #3).

### 4.2 RQ2: What Experiences, If Any, Appear to Shape Scientists’ Beliefs About Strategic Communication Help

The way that interviewees talked about their experiences receiving and giving help (RQ2) appeared to be consistent with our RQ1 results pointing to the idea that scientists fail to seek help with strategy because (a) such help is RARELY TOP‐OF‐MIND and (b) think of communication largely in terms of science PROMOTION. Specifically, as will be discussed, scientists’ descriptions of their experiences typically focused on positive or negative experiences with tactical issues (e.g., weak writing), rather than strategic issues (e.g., unclear goals). We discuss both positive and negative experiences, in turn.

On the positive side, one of the most frequent types of positive experiences that interviewees described was help sharpening or simplifying a message (coded as *positive experience—help focusing*). One interviewed scientist demonstrated such benefit beliefs in talking about working with one of their scientific societies on legislative efforts.

They'll often be telling us “Okay, that's great, but you need three sentences, and no more,” and “what are the highlights,” and they actually have helped us, you know, take what we want to say, but distill it down and refine it to make it a one pager, and hit the right key issues. FG8_sci_

Another top positive experience that respondents described was simply the chance to build relationship with a professional communicator (coded as *positive experience—extended relationship)*, but a key element for why such relationships were seen as beneficial was because they enabled greater accuracy or deeper storytelling. Similarly, making communication compelling (coded *positive experience—engaging or fun*) was also mentioned by several respondents. Few respondents specifically indicated that they valued deeper relationships between scientists and communicators because it helped them be more strategic. Instead, these positive experiences seemed to reflect the overall theme that scientists APPRECIATE any help they can get.

Descriptions of negative experiences also typically focused on experiences related to tactical‐level activities. This included several cases where scientists indicated that they believed that a communication professional wasted their time by not being prepared, misquoted them, or was just generally unskilled. Each of these was mentioned by about half of the interviews or focus group interviews (coded as *negative experience—waste of time*, *misquoted*, and *unskilled*). These seemed consistent with the theme that many of the conversations framed communication in terms of PROMOTION inasmuch as the negative experiences shared were generally about missed opportunities to promote research. We did not code any negative experiences related to colleagues’ disapproval about seeking communication support.

As suggested above, a counter‐narrative (i.e., negative/deviant cases) to the theme of strategy being RARELY TOP‐OF‐MIND was the finding that several interview subjects mentioned a desire to have someone on their communication team that better understood audiences. Such interview subjects often appeared to build on experience in which there were missed opportunities or difficulties communicating with communities who are often marginalized in science and may thus face added risks (i.e., Native/Aboriginal groups, people of color). For example, one respondent said the following:

I would want somebody who has some kind of like grounding or training with just like social justice and type work. That's something that has become more important for us to make connections with people and like, getting people in the communities like people who are living in poverty, or who are living in areas that are maybe under resourced, getting them to care about my research, like, why should they care, and it's been really helpful to have people have some kind of grounding as other ones other jobs. FG10_sci_

A smaller number of conversations (a bit less than half) similarly indicated a desire to have a social scientist on their team.

The biggest mistake people make in trying to communicate is they don't take enough time to think about their audience, who their audience is and where they're coming from. … And so, I would think a sociologist, or certainly some sort of social scientist … who understands people as receivers of this information and how to how to work and resonate with them. FG6_sci_

That being said, even in this quote is a sense that understanding audiences is a path to more efficient science PROMOTION rather than a desire to think strategically about goals and communicate *with* such audiences to achieve mutually beneficial outcomes. Further, social scientists were rarely the first people mentioned.

#### 4.2.1 Summary of RQ2 Findings

Overall, our sense was the positive and negative experiences we heard from interviewed scientists (and communication professionals) focused largely on tactical issues such as either poor or weak communication skills. This seemed consistent with the theme that issues of strategy such as goal setting, evidence‐based planning, and evaluation were RARELY TOP‐OF‐MIND for interviewees. Equally, these experiences tended to focus around missed opportunities to engage in science PROMOTION. Even still, however, interviewees seemed to APPRECIATE any opportunity to experience collaboration around communication. As with RQ1, these results continued to suggest to us that the primary reason that scientists are not asking for strategic communication help is because, per Reason #1 described above, they do not have a background that would make asking for strategic help especially likely. Issues of resources—Reason #2, above—likely exacerbate the challenge. We did not see substantial evidence that interview subjects had wide‐ranging negative beliefs about the trustworthiness of communicators, and thus, we do not believe Reason #3, above, is consistent with what we heard.

## 5 Discussion

One thing we noted in asking US‐based scientists to talk about communication was that they often noted that few people had ever invited them to talk about communication goals in substantive ways. By substantive, we mean the type of conversations that communication strategists in the private sector lead and/or the type of conversations that social scientists who study communication effects might have when deciding how to design a communication effort. There were exceptions, such as an early focus group interview with two practitioners (FG1_pro_) with deep backgrounds in working with diverse audiences. However, most conversations around experiences and potential hires, for example, quickly turned to discussions around how to do better broad science PROMOTION. Few conversations focused on specific communication goals or evidence‐based strategies to achieve those goals. Prompting such conversations typically required a substantial push from the interviewer. This experience underlies our identification of the theme that communication strategy is RARELY TOP‐OF‐MIND. It is worth further recalling that the people who agreed to be interviewed generally had some interest in communication and thus seem somewhat more likely than less‐interested scientists to have had experiences that pushed them to think more deeply about communication strategy.

Looking forward, this project suggests to us that anyone interested in enhancing the US scientific community's ability to think strategically around communication needs to look for ways to provide scientists *and* communication practitioners working in places such as universities with positive experiences with thinking about goals and how to achieve them. In this regard, we did not see substantial evidence that efforts by scholars—whether Besley and Dudo (2022) or groups such as the National Academies of Sciences, Engineering, and Medicine (2017)—to provide ways of thinking about science and risk communication more intentionally have yet had a substantial impact on how most of our interviewees discussed communication, at least in the US and in the context of environmental science. While not a theoretical problem, such findings challenge the utility of past efforts to promote a practical science of science communication (Jamieson et al. 2017).

On the other hand, what we heard makes us confident that many US‐based scientists would be willing to entertain more discussion of communication strategy. We base this view on our main finding that the people with whom we spoke tended to APPRECIATE the help they are receiving and seemed eager for more, deeper help.

One basic type of experience to provide scientists with might, at minimum, include more training that describes strategic processes for activities such as goal setting and strategy development. Although the data may be outdated, one interview‐based study of training programs suggests that they focus largely on skills (Dudo et al. 2021). Similarly, a more recent overview of programs suggested that less than a fifth of training programs address goal development, and only about a 10th addresses evaluation. In contrast, almost half address written and/or oral communication (Muindi and Luray 2023).

A more substantive type of experience that could be helpful is to provide scientists with the experience of working with a strategist either individually or as part of a group. Individual support might look like meeting with someone at various points in career, research program, or specific research project to talk through specific priorities (i.e., audience‐specific behavioral goals) for communication and then identify reasonable steps that might help achieve those goals. Ideally, such conversations might occur in the context of a group of scientists who share goals such that they can share time and other resources in ways that make goal achievement more feasible. Such an approach would be consistent with the increasing attention that scholars are putting on science and risk communication that originates from scientific organizations, rather than individual scientists (Entradas et al. 2020; Schäfer and Fähnrich 2020).

Other potential research pathways could include exploring scientists’ experiences in other countries and in more hierarchical organizational contexts. It should be expected, for example, that scientists working within government agencies with substantial communication mandates may have different experiences from university researchers who have substantial autonomy in making decisions about how to communicate.

## Author Contributions

**Sarah Garlick**: Conceptualization; Funding Acquistion; Investigation; Writing – Reviewing and Editing. **John C. Besley**: All stages. **Alexandra Benitez Gonalez**: Investigation; Data Curation; Writing – Reviewing and Editing.

## Funding

This material is based upon work supported by the National Science Foundation under Grant No. 2228259. Any opinions, findings, and conclusions or recommendations expressed in this material are those of the authors and do not necessarily reflect the views of the National Science Foundation.

## Supporting information




**Supporting Information**: risa70311‐supp‐0001‐SuppMat.docx

## Data Availability

Research data are not shared due to a need to ensure participant confidentiality.
