# Supplementary material for: Scientists’ Experiences With Getting Help Making Strategic Communication Choices
Source: Risk Anal. 2026 Jul 7;46(7):e70311. doi: 10.1111/risa.70311 (PMC13338910; doi:10.1111/risa.70311)
Supplement: Supplementary file 1 — Supporting Information: risa70311‐supp‐0001‐SuppMat.docx [file RISA-46-0-s001.docx]

Supplemental Material

Supplemental table A. Moderator/interview guide (Condensed)

| **Concept/Idea** | **Potential Questions** |
| --- | --- |
| 1. Top-of mind experiences working with communication professionals. 2. Quantity? 3. Quality?   (10-15 minutes) | - (Ask to think/jot down ideas)* - To start, does anyone [Do you] have a top-of-mind, positive experiences they [you] can share about working with a communication professional/ scientist? (REPEAT) - What lessons do you think we could take from that type of experience? - Does anyone [Do you] have a top-of-mind, negative experiences they [you] can share about working with a communication professional/ scientist? (REPEAT) |
| 1. Factors that might make scientists more likely to collaborate with communication professionals for help communicating.   (10-15 minutes) | - When do you think scientists should collaborate with communication professionals? - What kinds of communication activities do you think scientists should be able to do on their own? - What kinds of discussions have you had about how and when to ask for communication help? |
| 1. Perceptions about what would make a good communication support team.    1. What are the most important skills needed?    2. Who should a communication team report to? 2. How should it be managed?   (10-15 minutes) | - If you were asked to hire professionals who would help scientists in your department improve public engagement programs and activities, what kind of competencies do you think you’d want to look for? - If you were asked to help build a team of three of four communication professionals, what types of skills would you look for?   Potential probe: What’s the relative importance of scientific knowledge about the subject versus communication expertise?  Potential probe: What role for a strategist/someone to help with setting goals and creating a strategy to achieve those goals on your potential team? |
| 1. Perceived need for resource allocation   (10-15 minutes) | - How do you think public engagement activities should be funded? - To what degree do existing budgets for public engagement activities allow for scientist-professional collaborations?   Potential probes  If a scientist is proposing a $250K, 3-year grant, what’s an appropriate amount to budget for communication/ engagement? What do you hope that’d pay for?   - How about if it’s a $1 million grant? What do you hope that’d pay for? - [For scientists] What factors would make you allocate more money for public engagement? Less? |
| 1. Direct question   (10-15 minutes) | - What do you think is the biggest problem/opportunity right now when it comes to providing scientists with what they need to communicate/engage effectively and efficiently? |
| 1. Direct question   (10-15 minutes) | - Our task is to come up with a research program that helps us understand what factors make it more or less likely that scientists will both fund and rely on communication experts to help them strategize around science communication.   - Building on today’s conversation, are there any potential research questions you have in this area that you think we should prioritize? |

Notes: *Focus group only.
